# New insights in the expression of stromal caveolin 1 in breast cancer spread to axillary lymph nodes

**DOI:** 10.1038/s41598-021-82405-y

**Published:** 2021-02-02

**Authors:** Cristian Scatena, Giovanni Fanelli, Giuseppe Nicolò Fanelli, Michele Menicagli, Paolo Aretini, Valerio Ortenzi, Sara Piera Civitelli, Lorenzo Innocenti, Federica Sotgia, Michael P. Lisanti, Antonio Giuseppe Naccarato

**Affiliations:** 1 Division of Pathology, Department of Translational Research and New Technologies in Medicine and Surgery, University of Pisa, 56126 Pisa, Italy; 2 Department of Laboratory Medicine, Pisa University Hospital, Anatomia Patologica 1 Universitaria, 56126 Pisa, Italy; 3 Fondazione Pisana per la Scienza, 56017 Pisa, Italy; 4 Translational Medicine, University of Salford, Greater Manchester, M5 4WT UK

**Keywords:** Breast cancer, Cancer microenvironment, Metastasis, Cancer

## Abstract

Recent evidence suggests that a loss of expression of caveolin in the stromal compartment (sCav-1) of human invasive breast carcinoma (IBC) may be a predictor of disease recurrence, metastasis and poor outcome. At present, there is little knowledge regarding the expression of sCav-1 at the metastatic sites. We therefore studied sCav-1 expression in IBCs and in their axillary lymph nodes to seek a correlation with cancer metastasis. 189 consecutive invasive IBCs (53 with axillary lymph node metastases and 136 without) were studied by immunohistochemistry, using a rabbit polyclonal anti-Cav-1 antibody. In IBCs sCav-1 was evaluated in fibroblasts scattered in the tumor stroma whereas in lymph nodes sCav-1 was assessed in fibroblast-like stromal cells. For the first time, we observed a statistically significant progressive loss of sCav-1 from normal/reactive axillary lymph nodes of tumors limited to the breast to metastatic axillary lymph nodes, through normal/reactive axillary lymph nodes of tumors with axillary metastatic spread. These data indicate that Cav-1 expressed by the stromal compartment of lymph nodes, somehow, may possibly contribute to metastatic spread in IBC.

## Introduction

Breast cancer is a heterogeneous disease, the most frequent malignant tumor in women worldwide, with more than 2 million new cases and nearly 630.000 estimated deaths in 2018^1^**.** Studies have traditionally focused on elucidation of the events arising in the cancerous epithelial cells with little attention to the role of the surrounding stroma or microenvironment^2^. At present, fibroblasts, blood vessels and extracellular components are thought to be key regulators of cancer progression. Indeed, modified fibroblasts, the so-called “cancer associated fibroblasts” (CAFs), modulate extracellular matrix degradation^3^ and regulate proliferation of the epithelial cancer cells within the primary tumor site, via the production of numerous growth factors and cytokines, including HGF, EGF, IGFs, IGFBPs, b-FGF and TGF-β^4^. The tumor microenvironment likely plays a role also at metastatic sites, to re-establish the conditions that disseminated tumor cells (DTCs) require for growth^5^. The term “metastatic niche” has been coined to refer to this phenomenon and has been described for visceral secondary sites but also for lymph nodes: here, fibroblast-like stromal cells, together with bone marrow derived cells, play a crucial role to form these permissive compartments^6^. At present, the precise complex mechanisms through which the stroma orchestrates tumor progression are not fully elucidated. In recent decades, increasing attention has been paid to the expression of caveolin-1 (Cav-1) in the tumor microenvironment. Cav-1 is the essential constituent protein of caveolae, specialized flask-shaped invaginations of the plasma membrane, that act as signal transduction hubs by providing compartmentalization of intracellular signaling molecules^7^. Cav-1 is expressed on the cell membrane of endothelial cells, adipocytes, smooth muscle cells and type 1 pneumocytes. Inside caveolae, Cav-1 binds to G-protein subunits, tyrosine kinases and endothelial nitric oxide synthase, thus regulating several signaling pathways^8^. Mouse models studies demonstrated that caveolins play an important role in a number of human diseases such as atherosclerosis, muscular dystrophy, diabetes and cancer^9^. The role for this protein in the process of cell transformation was reported in 1989, when Cav-1 was identified as a substrate for v-Src in transformed chicken embryo fibroblasts^10^. Later research suggested Cav-1 gene as tumor suppressor as well as proto-oncogene: downregulation is associated to colon and ovarian carcinomas or sarcomas instead upregulation to prostate, lung, esophageal, bladder and papillary thyroid carcinomas^11^.

In breast cancer, Cav-1 gene has been suggested as tumor suppressor^12^; however, aggressive breast cancer subtypes overexpress Cav-1 protein, supporting an oncogenic role^13^. Thus, the role of Cav-1 in tumorigenesis remains contentious. Interestingly, a number of recent studies have investigated the role of Cav-1 in the tumor microenvironment, describing stromal Cav-1 (sCav-1) as a marker of the CAF phenotype in human breast cancer^14^. In particular, the decreased expression of sCav-1 is associated with poor prognosis^15–17^. Loss of sCav-1 also predicts poor outcome^18^, in particular in the subgroup of basal-like breast cancers^19^. In addition, a striking correlation between loss of sCav-1 and early progression from in situ to invasive breast carcinoma has recently been reported^20^. However, most of the studies have concentrated so far on sCav-1 in primary tumor and there is little knowledge about the expression of sCav-1 at the metastatic sites, in breast cancer or in other tumor models. The aim of this study was to investigate the expression of Cav-1 in: i) the stromal compartment of axillary lymph nodes of breast cancer patients, to seek a correlation with cancer metastasis; ii) in the stroma of invasive breast carcinomas (IBCs), to confirm data from published studies.

## Results

### Expression of sCav-1 in lymph nodes

**Figure 1:**
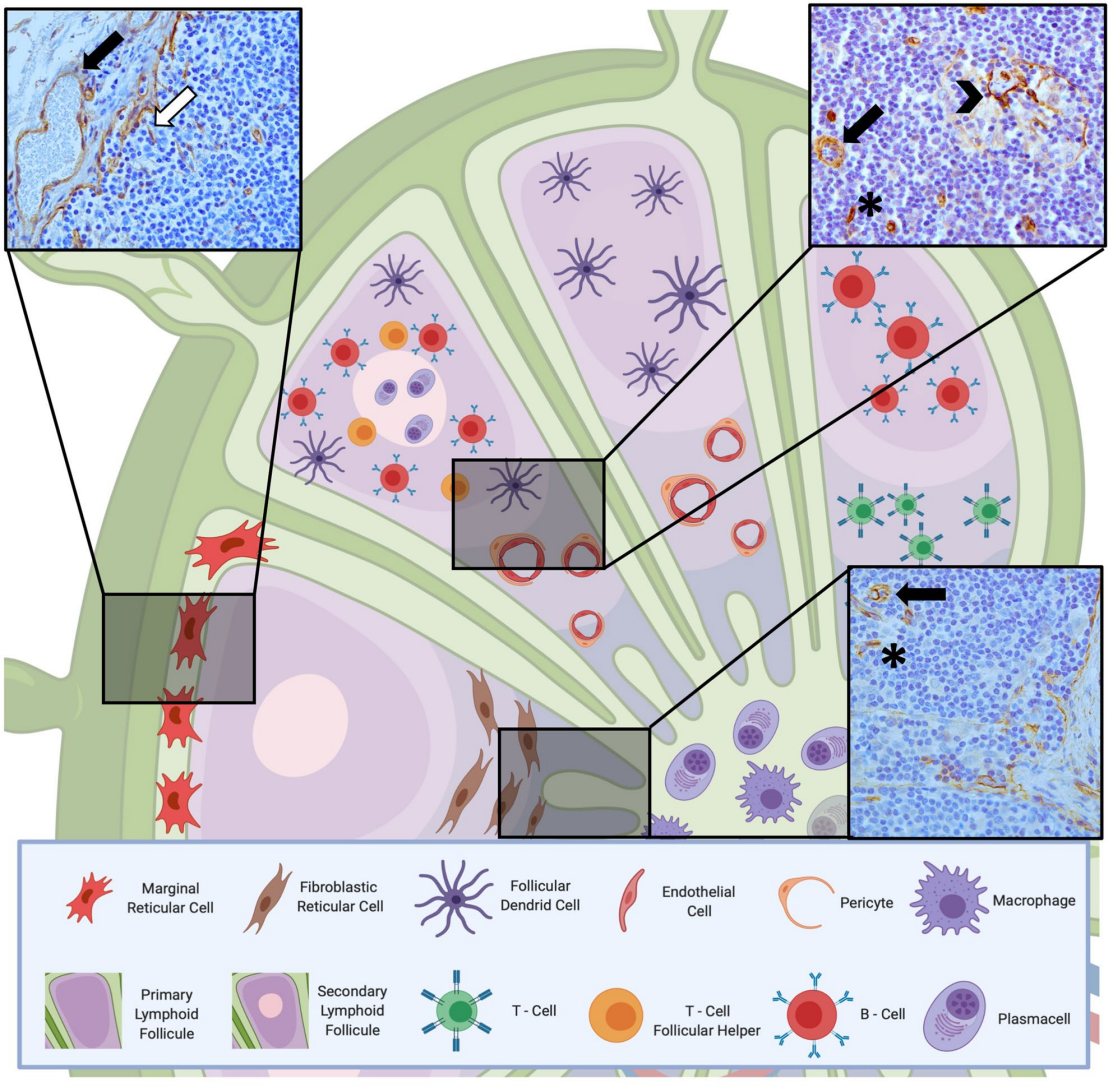
Illustration of fibroblast-like stromal cells in a lymph node. In the insets three examples of sCav-1 positive cells in a normal lymph node. Together with endothelial cells (black arrow) and pericytes, fibroblast-like stromal cells, such as marginal reticular cells (white arrow) under the subcapsular sinus, fibroblastic reticular cells in the paracortex/T cell zone (*), and follicular dendritic cells in lymphoid follicles (arrowhead) constitute the stromal compartment of a lymph node. All IHC images magnification: 200x. Created with BioRender.com.

We evaluated the expression of sCav-1 in the stromal compartment of lymph nodes (see Fig. 1) which is composed of: (i) fibroblast-like stromal cells, a heterogenous population that looks like fibroblasts (with spindle shape cytoplasm and elongated nucleus) which encompasses marginal reticular cells under the subcapsular sinus and fibroblastic reticular cells in the paracortex/T cell zone; (ii) follicular dendritic cells in lymphoid follicles (with several cytoplasmic extensions/dendrites); (iii) endothelial cells and (iv) pericytes. These cells have distinctive locations and different functions, some of which not yet fully elucidated.

According to our knowledge, to date a specific immunomarker for each fibroblast-like stromal cells is lacking, and their identification mostly relies on location and morphologic features by which they can be easily distinguished from lymphocytic cells, plasma cells and monocytes/macrophages.

**Figure 2:**
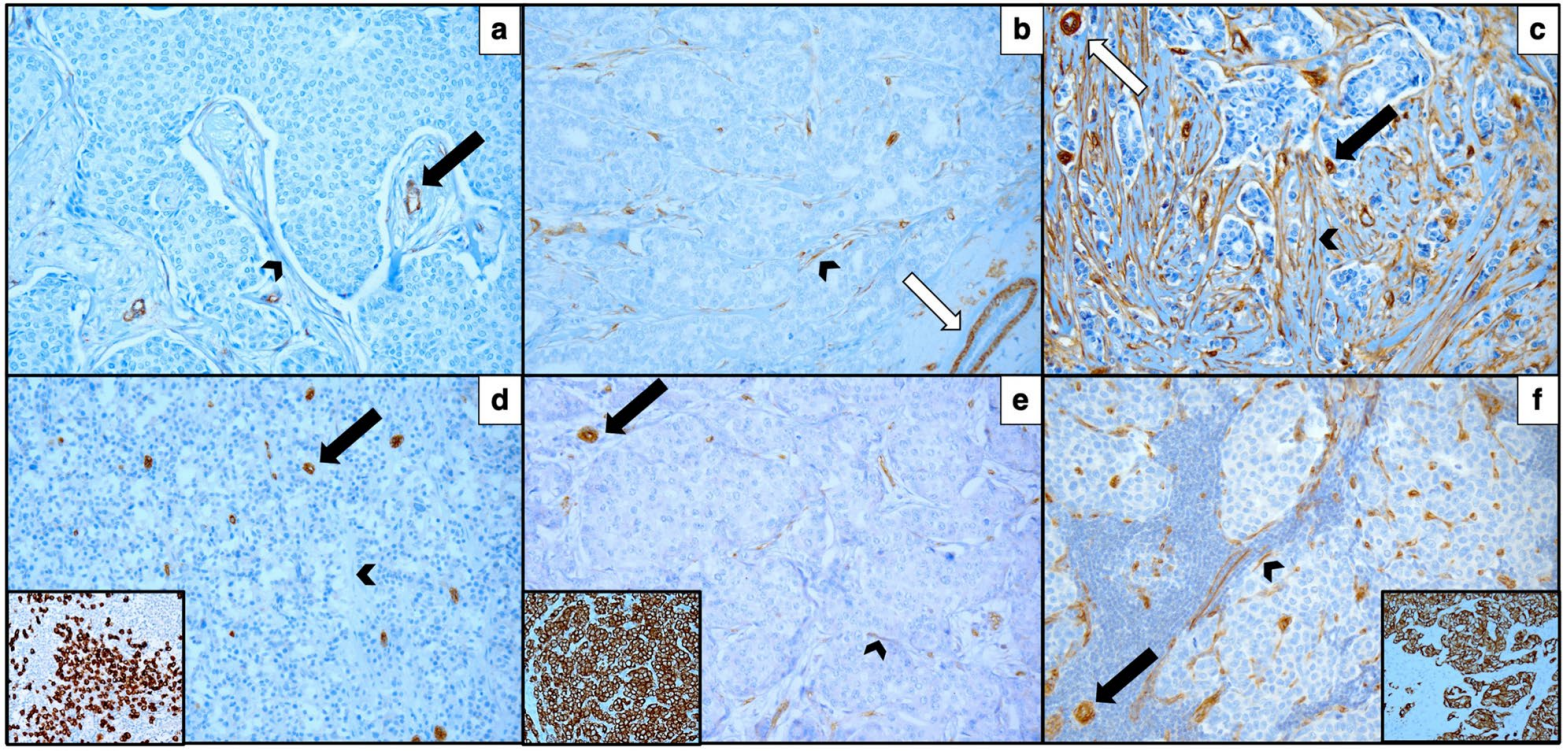
Semiquantitative score of sCav-1 expression. **(a)** Absent (Score 0), **(b)** weak (Score 1) and **(c)** strong (Score 2) expression of sCav-1 in cancer associated fibroblasts (arrowhead) in primary invasive breast cancer. **(d)** Absent (Score 0), **(e)** weak (Score 1) and **(f)** strong (Score 2) expression of sCav-1 in fibroblast-like stromal cells (arrowhead) within metastatic lymph nodes; in the insets Pan-CK expression in metastatic cells. Internal positive controls: endothelial cells (black arrow) and myoepithelial cells (white arrow). All images magnification: ×200.

Among axillary lymph nodes of IBC cases, 119 (65.4%) showed expression of sCav-1 in fibroblast-like stromal cells scattered within lymphoid tissue (see Fig. 2d,e,f and Table 1 for details).

**Table 1:** Distribution of sCav-1 expression among IBC subtypes and corresponding lymph nodes (LN).

	Score 2	Score 1	Score 0	Total IBC (LN)
IBC-NST(LN)	48(15)	86(51)	6(44)	140(110)
w.d.(LN)	16(2)	27(7)	2(0)	45(9)
m.d.(LN)	11(5)	32(9)	4(0)	47(14)
p.d.(LN)	21(8)	27(35)	0(44)	48(87)
ILC(LN)	19(23)	28(30)	2(19)	49(72)
Total IBC(LN)	67(38)	114(81)	8(63)	189(182)

*IBC-NST* invasive carcinoma of no special type, *w.d.* well differentiated, *m.d.* moderately differentiated, *p.d.* poorly differentiated, ILC invasive lobular carcinoma.

**Figure 3:**
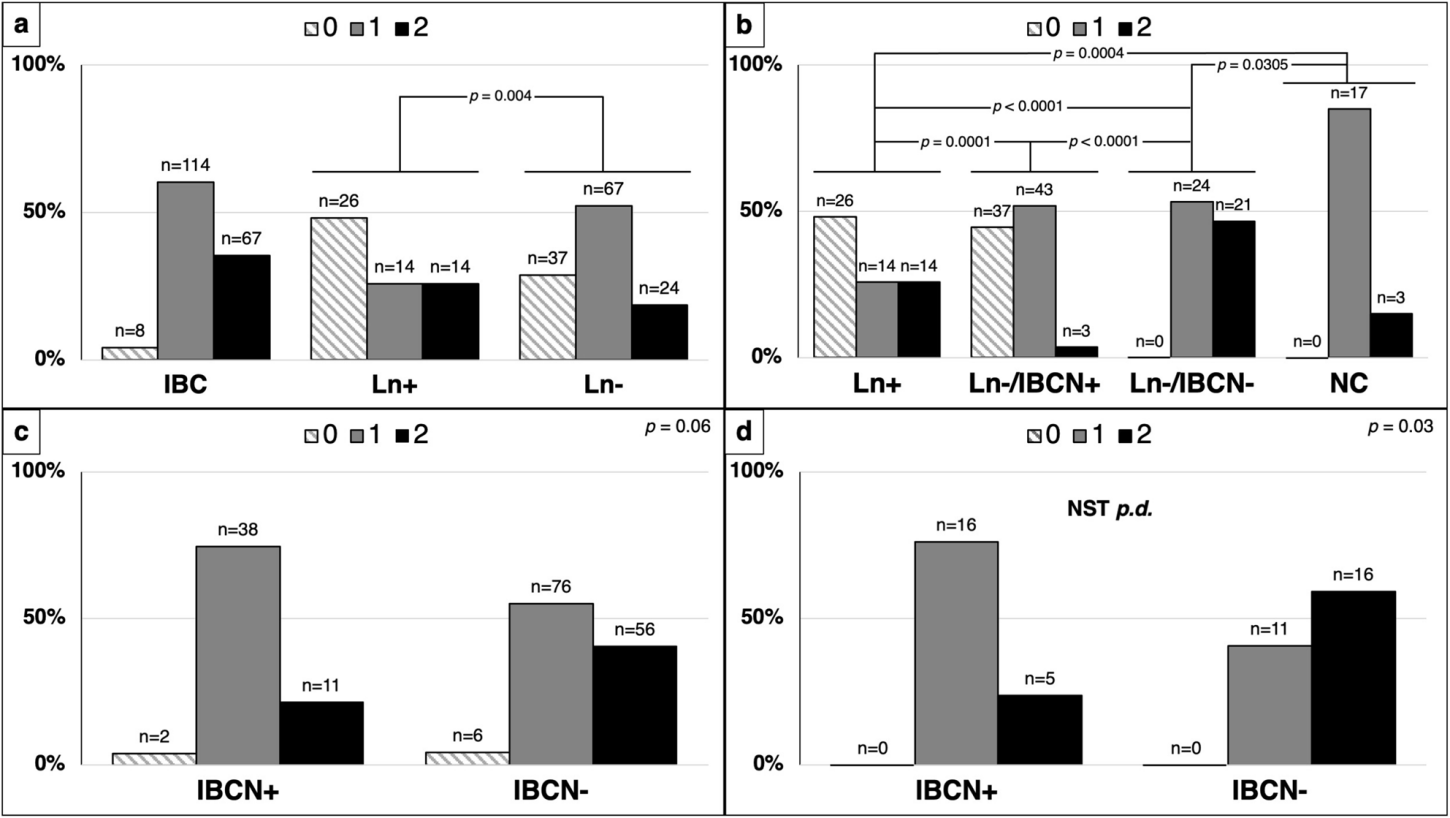
**(a)** Expression of sCav-1 in primary IBCs and lymph nodes. In contrast to primary IBCs (regardless the nodal status), a high number of metastatic lymph nodes (Ln +) showed score 0 staining for sCav-1. Ln + demonstrate significant lower expression of sCav-1 than lymph nodes without metastasis (Ln−). **(b)** sCav-1 expression in lymph nodes. From right to left, sCav-1 is increasingly lost from lymph nodes with reactive hyperplasia from cases without nodal metastatic disease (Ln−/IBCN−), through non-metastatic lymph nodes from cases with nodal metastatic disease (Ln−/IBCN +), to Ln + ; lymph nodes of patients with no history of cancer (NC) show undoubtedly high expression of sCav-1, comparable to Ln−/IBCN−, even if slightly lower. **(c)** Correlation of sCav-1 expression in primary IBCs and nodal status. The histograms show how primary tumors with nodal metastasis (IBCN +) demonstrate lower expression of sCav-1 than primary tumors without metastasis (IBCN−), regardless the grade. **(d)** Correlation of sCav-1 expression in primary poorly differentiated (*p.d.*) IBCs of no special type (NST) and nodal status. The histograms show how IBCN + demonstrate statistically significant lower expression of sCav-1 than IBCN− in the NST *p.d.* subgroup. sCav-1 staining was scored semi-quantitatively as 0, 1 and 2 (see text).

All normal/reactive axillary lymph nodes of patients included as negative controls (NC) were positive: 17 with score 1 and 3 with score 2 (see Fig. 3b). Instead, a large number of metastatic axillary lymph nodes (Ln +) (26 out of 54, 48%) showed a loss of sCav-1 expression: sCav-1 was significantly less expressed in metastatic axillary lymph nodes (Ln +) than in normal/reactive axillary lymph nodes (Ln−) (p-value = 0.0042) (see Fig. 3a for details). In particular, the difference was strikingly more evident between metastatic axillary lymph nodes (Ln +) and normal/reactive axillary lymph nodes of tumors limited to the breast (Ln−/IBCN−) (p-value < 0.0001); normal/reactive axillary lymph nodes of tumors with axillary metastatic spread (Ln−/IBCN +) showed an in-between sCav-1 profile: higher if compared to metastatic axillary lymph nodes (Ln +) (p-value = 0.0001) and lower if compared, instead, to normal/reactive axillary lymph nodes of tumors limited to the breast (Ln−/IBCN−) (p-value < 0.0001) (see Fig. 3b). Axillary lymph nodes of NC showed a sCav-1 expression higher than normal/reactive axillary lymph nodes of tumors with axillary metastatic spread (Ln−/IBCN +) (p-value = 0.0004) and slightly lower than normal/reactive axillary lymph nodes of tumors limited to the breast (Ln−/IBCN−) (p-value = 0.0305, Fig. 3b). These results indicate that axillary lymph nodes express high levels of sCav-1, but metastatic axillary lymph nodes (Ln +) express lower levels of sCav-1 compared to normal/reactive axillary lymph nodes (Ln−); intriguingly, among normal/reactive axillary lymph nodes those close to metastatic lymph nodes (Ln−/IBCN +) express lower levels of sCav-1 if compared to those from tumors limited to the breast (Ln−/IBCN−).

### Expression of sCav-1 in primary breast tumors

sCav-1 expression has been evaluated in all the 189 primary IBC cases examined. Myoepithelial cells and endothelium exhibited strong staining in all the samples examined, as expected, indicating a proper immunohistochemical reaction (see Fig. 2a–c).

In particular, 181 primary tumors (95.8%) [of which 134 cases were IBCs of No Special Type (IBCs-NST) (95.7%) and 47 cases were Invasive Lobular Cancers (ILCs) (95.9%)] stained positive for sCav-1 (67 with score 2, 114 with score 1). 8 cases (4.2%) were negative (score 0).

Of the 189 cases, sCav-1 expression was lower in those tumors with axillary metastatic spread (IBCN +) compared to those limited to the breast (IBCN−), with a p-value close to statistical significance (p = 0.0610, see Fig. 3c).

In the attempt to stratify sCav-1 expression among the different subtypes, we have found: i) no significant difference between IBCs-NST and ILCs; ii) among IBCs-NST, a statistically significant correlation between loss of sCav-1 and presence of lymph node metastases in poorly differentiated carcinomas (p-value = 0.03, see Fig. 3d).

### Recurrence rate and sCav-1 expression in primary IBCs

We have collected patients’ clinical data up until 20th of September 2020 (see Supplementary Table S1). According to these data: (i) 28 patients have been lost to follow-up (the loss to follow-up rate was quite low: ~ 15%); (ii) 13 patients with IBC-NST and 7 patients with ILC have relapsed; the total recurrence rate was 13% and, in particular: 12% for IBC NST and 16% for IBC ILC.

Interestingly, even if only ~ 4% of primary IBCs totally lacked the expression of sCav-1 (score 0), we have found that ~ 74% of the relapsed cases had only a weak expression of sCav-1 (score 1) regardless the histotype (10 of 13 were IBC-NST and 5 of 7 were ILC) (see Supplementary Table S2).

In addition, all relapsed cases were grade 3 or 2; none grade 1 tumors relapsed.

In conclusion, our data are in line with the literature; indeed, sCav-1 loss in primary breast cancer is a negative prognostic factor and is common in high grade breast cancer^19^.

### Potential pitfalls

Selected samples represented a relatively small consecutively series in a mono-institutional setting; this could represent a limitation to draw statistically significant conclusions about the correlation between sCav-1 expression and patients outcomes, even if this is not the main aim of the present article. Moreover, this could be the reason for the lower rate of primary IBC sCav-1 negative in respect to the literature^18^.

This research article would present preliminary results that support new insights on the potential role of sCav-1 in lymph node metastatic niche, paving the way for further investigations. However, data need to be validated in a wide well-studied and well-annotated multicentric cohort with a long term follow-up, in order to confirm envisaged scenarios.

## Discussion

To date, the relationship between Cav-1 and cancer remains controversial^11^. In the past, human studies extensively focused on the role of Cav-1 in epithelial cancer cells reporting a significant increase in Cav-1 expression in malignant lesions than benign breast tissue^21–25^. In recent decades, increasing attention has been paid to the expression of Cav-1 in the tumor stroma where its loss predicts poor outcome^15–17^. In the present study, immunohistochemistry performed on IBCs primary tumors demonstrated a considerable trend toward significance between a loss of sCav-1 and presence of lymph node metastases. Nevertheless, statistical significance is achieved when only poorly differentiated NST IBCs are considered. These data are in accordance to those from the literature, where a strong association was observed between a loss of sCav-1 and the presence of lymph node metastases in triple negative tumors^19^. This suggests that especially IBCs with aggressive behavior may be sensitive to sCav-1 regulation in their progression into metastatic disease. In contrast, at present there is still little knowledge regarding the expression of sCav-1 at metastatic sites. Here, DTCs survive and grow as metastasis in presence of a supportive tumor stroma, the so-called “metastatic niche”, whose evolution thus recapitulates stromal progression in the primary tumor^5^. The formation of the metastatic niche can also be remotely induced by primary tumors before the arrival and establishment of DTCs: for such cases, the term “pre-metastatic niche” has been coined^26^. This phenomenon has been described also for lymph nodes, where fibroblast-like stromal cells, together with bone marrow derived cells, play a crucial role to form these permissive compartments^6^. The present study investigated the expression of sCav-1 in the tumor stroma of IBCs and in their corresponding axillary lymph nodes, in order to define any possible correlation with cancer metastasis. In accordance to Alevizos et al*.*, sCav-1 was significantly less expressed in lymph node metastases than lymph nodes with reactive hyperplasia, of metastatic or non-metastatic cases, confirming that low expression of Cav-1 in fibroblast-like stromal cells of axillary lymph nodes correlates with high metastatic potential^27^. Intriguingly, Celus et al*.* recently demonstrated in mouse models of lung and breast cancer metastasis that Cav-1 inhibition in the so-called metastasis-associated macrophages (MAMs) drives metastatic growth favoring angiogenesis by the restriction of vascular endothelial growth factor A/vascular endothelial growth factor receptor 1 (VEGF-A/VEGFR1) signaling and its downstream effectors at the metastatic site^28^. A similar mechanism may be involved in humans. For the first time to our best knowledge, we described a statistically significant progressive loss of sCav-1 from normal/reactive axillary lymph nodes from tumors limited to the breast (Ln−/IBCN−) to metastatic axillary lymph nodes (Ln +), through normal/reactive axillary lymph nodes of tumors with axillary metastatic spread (Ln−/IBCN +). These data may suggest that Cav-1 expressed by the stromal compartment of lymph nodes, somehow, may play a possible role in establishing the pre-metastatic niche in IBC. The lower expression of sCav-1, observed not only in lymph node metastases but also in close lymph nodes with reactive hyperplasia (Ln−/IBCN +), may hint at the possible role of the primary tumor or of the DTCs in the microenvironmental changes of loco-regional lymph nodes, contributing to the formation of these permissive compartments. In order to better investigate which of the two—the primary tumor or DTCs—were more likely to be involved, we included lymph nodes from patients with no history of cancer but with reactive hyperplasia (as negative control, NC), which showed consistently high expression of sCav-1. This would hint at the possibility that sCav-1 in lymph nodes might be modulated by DTCs, present in the same lymph node or in close vicinity, and not remotely by primary tumors at distance. Indeed, if the primary tumor exerted a permissive effect for the infiltration and growth of DTCs in the axilla, lymph nodes from patients with tumors limited to the breast (Ln−/IBCN−) should have shown a lower expression of sCav-1 than NC. Instead, the expression of sCav-1 in NC resulted even lower than axillary lymph nodes from patients with tumors limited to the breast (Ln−/IBCN−). According to this hypothesis, DTCs might in turn provide the permissive pre-metastatic niche in close lymph nodes. In 2005, Kaplan et al*.* firstly demonstrated that bone marrow-derived hematopoietic progenitor cells that express vascular endothelial growth factor receptor 1 (VEGFR1) home to tumor-specific pre-metastatic sites and form cellular clusters before the arrival of tumor cells^6^. The authors also showed that VEGFR1 + cells express VLA-4 (also known as integrin α4β1), and that tumor-specific growth factors upregulate fibronectin—a VLA-4 ligand—in resident fibroblasts, providing a permissive niche for incoming tumor cells. According to this, recent evidence suggest that tumor secreted factors modify perivascular cells to create a pro-metastatic environment enriched in fibronectin^29^. In 2008, Shi and Sottile showed how Cav-1-dependent β1 integrin endocytosis is a critical regulator of fibronectin turnover; in particular, they demonstrated that Cav-1 downregulation reduces both fibronectin and β1 integrin endocytosis^30^. Within this scenario, a loss of expression of sCav-1 in lymph nodes may contribute to the localized deposition of fibronectin, providing permissive compartments for DTCs. In addition, sCav-1 has been recently described to promote the ability of dendritic cells (DC) to reach the lymph nodes and initiate CD8 + T cells response. Indeed, Oyarce et al*.* have reported that caveolin-1 expression increases upon maturation in DCs and promotes their migration to lymph nodes favoring the activation of CD8 + T cells. CD8 + T cells response represents a key mechanism against pathogens but also against solid tumors. To confirm this hypothesis the Authors demonstrated a reduction in CD8 + T cell response and antitumor protection in mice that received CAV−/− DCs^31^. These data strongly suggest that a loss of expression of sCav-1 in lymph nodes may inhibit anti-tumor CD8 + T cells, thus favoring the initiating capacity of incoming DTCs. Finally, immunohistochemical studies of human breast cancer showed that specimens lacking sCav-1 overexpress key enzymes of the glycolytic pathway, such as PKM2 and LDH^32,33^, thus generating, in the pre-metastatic niche, high quantities of energy metabolites (i.e. lactate and pyruvate) to feed the arriving DTCs. As a result, the loss of expression of sCav-1 in axillary lymph nodes may promote successful seeding of arriving cancer cells via at least three fascinating mechanisms: the increase of fibronectin deposition, the inhibition of anti-tumor CD8 + T cells and the increase of energy metabolites availability. Further studies are needed to better understand either the mechanisms through which a loss of sCav-1 may contribute to the regulation of metastatic niche formation or the mechanisms through which cancer cells induce the downregulation of sCav-1, a process currently still largely poorly understood.

In conclusion, our findings, in addition to supporting data from the literature, provide further and new evidence of a correlation between sCav-1 expression in lymph nodes and lymph node metastases, suggesting a possible role in the establishment of the pre-metastatic niche in breast cancer.

Further analyses on larger cohorts of patients with adequate number of lymph node metastases are required to validate these preliminary data definitely; moreover, functional studies in vitro are needed to study in depth the biological mechanisms underlying these preliminary observations.

## Materials and methods

### Case selection

189 cases (53 with lymph node metastases and 136 without) were examined, from women with a median age of 57.55 years (range 31–89 years). All IBCs were classified according to 2019 World Health Organization (WHO) Classification into IBC-NST, graded according to the Nottingham Modification of the Bloom-Richardson system, and ILC (Table 2).

**Table 2:** Patients selection: sex, median age and distribution of different subtypes of IBC.

Sex	F
Median age	57.55 years
IBC-NST(N +)	140(39)
w.d	45(3)
m.d	47(15)
p.d	48(21)
ILC(N +)	49(14)
Total(N +)	189(53)

*F* female, *y* years, *IBC-NST* invasive breast carcinoma of no special type, *N +*  number of cases with lymph node metastasis, *w.d.* well differentiated, *m.d.* moderately differentiated, *p.d.* poorly differentiated, ILC invasive lobular carcinoma.

137 axillary lymph nodes were collected from 28 cases with axillary metastatic spread (IBCN +); among these, 54 displayed metastasis (Ln +), 83 did not (Ln−/IBCN +). In addition, 45 axillary lymph nodes were collected from other 28 cases with tumors limited to the breast (Ln−/IBCN−). Therefore, three groups of study interest were delineated (Table 3): (i) metastatic axillary lymph nodes (Ln +); (ii) normal/reactive axillary lymph nodes of tumors with axillary metastatic spread (Ln−/IBCN +); (iii) normal/reactive axillary lymph nodes of tumors limited to the breast (Ln−/IBCN−).

**Table 3:** Distribution of lymph nodes, through respective subtypes of IBC.

	Ln +	Ln−/IBCN +	Ln−/IBCN−	Total
IBC-NST	31	56	23	110
w.d	2	3	4	9
m.d	4	3	7	14
p.d	25	50	12	87
ILC	23	27	22	72
Total	54	83	45	182
	137			

*Ln +*  number of metastatic lymph nodes from cases with nodal metastatic disease, *Ln−/IBCN +*  number of non-metastatic lymph nodes from cases with nodal metastatic disease, *Ln−/IBCN−* number of lymph nodes with reactive hyperplasia from cases without nodal metastatic disease, *IBC-NST* invasive breast carcinoma of no special type *w.d.* well differentiated, *m.d.* moderately differentiated, *p.d.* poorly differentiated, *ILC* invasive lobular carcinoma.

Twenty normal/reactive axillary lymph nodes of patients admitted at our hospital for cardiac surgeries and with no history of cancer were included as negative controls (NC). All of the specimens studied were retrieved from the files of the Division of Surgical, Molecular, and Ultrastructural Pathology, University of Pisa (Italy) from January 2006 to January 2011.

sCav-1 was evaluated: (i) in lymph nodes, in fibroblast-like stromal cells classified as marginal reticular cells (below the sub-capsular sinus), fibroblastic reticular cells (of the paracortex/T cell zone) and follicular dendritic cells (of B follicles) (see Fig. 1); (ii) in IBCs, in fibroblasts scattered in the tumor stroma.

All investigations were conducted according to the principles expressed in the Declaration of Helsinki; this study was approved by the "Ethical Committee for the testing and evaluation of clinical study protocols in the North Wester Tuscany Area (CEAVNO)" (Prot. n: 14,835. Approval date: 11/04/2019). Written informed consent was obtained from each participant.

### Immunohistochemistry

Formalin-fixed, paraffin-embedded (FFPE) tissue blocks were cut into 4 µm thick sections. Slides were incubated with rabbit polyclonal anti-Caveolin-1 antibody (N20) (Santa Cruz CA, dilution 1:500) at 37 °C for 32 min (Ventana Benchmark XT staining system, Ventana Medical System-Roche) and developed in diaminobenzidine (DAB)—hydrogen peroxide for 10 min (ultraView Universal DAB kit, Ventana Medical System-Roche). Finally, sections were counterstained with hematoxylin and mounted. The staining was scored semi-quantitatively as 0 (negative, in stromal cells), 1 (diffuse weak or strong in less than 30% of stromal cells) and 2 (strong in 30% or more of the stromal cells)^20^. Both membranous or combined membranous and cytoplasmic staining was used for Cav-1 expression evaluation. Positive staining of myoepithelial cells or endothelium, known to be abundant in Cav-1, served as an internal positive control.

### Statistical analysis

The Chi-Square test was used to analyze the association between the different phenotypes and Cav-1 expression. All statistical analyses were performed by means of Medcalc for Windows (Version 12). A *p*-value of less than 0.05 was considered statistically significant.

## Supplementary Information

Supplementary Tables.
