# Supplementary material for: New insights in the expression of stromal caveolin 1 in breast cancer spread to axillary lymph nodes
Source: Sci Rep. 2021 Feb 2;11:2755. doi: 10.1038/s41598-021-82405-y (PMC7854652; doi:10.1038/s41598-021-82405-y)
Supplement: Supplementary file 1 — Supplementary Tables. [file 41598_2021_82405_MOESM1_ESM.pdf]

New insights in the expression of stromal caveolin 1 in breast cancer spread to axillary lymph nodes

Cristian Scatena, Giovanni Fanelli, Giuseppe Nicolò Fanelli, Michele Menicagli, Paolo Aretini, Valerio Ortenzi, Sara Piera Civitelli, Lorenzo Innocenti, Federica Sotgia, Michael P. Lisanti, and Antonio Giuseppe Naccarato

CASES	IBC-NST G1	IBC-NST G2	IBC-NST G3	ILC	Total
T_0	45	47	48	49	189
T^1	37	42	39	43	161
LOST TO FOLLOW-UP	8	5	9	6	28
RELAPSED	0	5	8	7	20

Supplementary Table S1. Relapsed and lost to follow-up cases stratified according to IBC subtypes.
IBC-NST, Invasive Breast Carcinoma of No Special Type; ILC, Invasive Lobular Carcinoma; G, Grade; T_0, time of first diagnosis; T_1, time of last clinical data collection (see text).

sCav-1 expression in primary relapsed IBCs at T_0	IBC-NST G1	IBC-NST G2	IBC-NST G3	ILC	Total
0	0	0	0	0	0
1	0	5	5	5	15
2	0	0	3	2	5

Supplementary Table S2. Relapsed cases stratified according to sCav-1 expression scores in primary IBCs .
IBC-NST, Invasive Breast Carcinoma of No Special Type; ILC, Invasive Lobular Carcinoma; G, Grade; T_0, time of first diagnosis.
